# *Campylobacter hepaticus*, the Cause of Spotty Liver Disease in Chickens: Transmission and Routes of Infection

**DOI:** 10.3389/fvets.2019.00505

**Published:** 2020-01-15

**Authors:** Canh Phung, Ben Vezina, Arif Anwar, Timothy Wilson, Peter C. Scott, Robert J. Moore, Thi Thu Hao Van

**Affiliations:** ^1^School of Science, RMIT University, Bundoora West Campus, Bundoora, VIC, Australia; ^2^Scolexia Pty Ltd., Moonee Ponds, VIC, Australia

**Keywords:** *Campylobacter hepaticus*, chicken, environment, epidemiology, spotty liver disease, transmission

## Abstract

The epidemiology of Spotty Liver Disease (SLD) was investigated by assaying 1,840 samples collected from layer chickens and the environment in poultry farms across Australia for the presence of *Campylobacter hepaticus*, the agent responsible SLD in chickens. A *C. hepaticus* specific PCR and bacterial culture were used. Results showed that birds could be infected with *C. hepaticus* up to 8 weeks before clinical SLD was manifested. In addition, birds could be infected long before laying starts, as young as 12 weeks old, but the peak period for SLD outbreaks was when the birds were 26–27 weeks old. *Campylobacter hepaticus* DNA was detected in motile organisms such as wild birds and rats and so these organisms may be vectors for *C. hepaticus* dissemination. Moreover, water, soil, mites, flies, and dust samples from SLD infected farms were also found to be PCR-positive for *C. hepaticus* DNA. However, it still remains to be determined whether these environmental sources carry any viable *C. hepaticus*. The indications from this study are that environmental sources are a likely transmission source of *C. hepaticus*. Therefore, biosecurity practices need to be strictly followed to prevent the spread of SLD amongst and between flocks. Also, a rapid, molecular detection method such as PCR should be used as to monitor for *C. hepaticus* presence in flocks before clinical disease is apparent, and therefore inform the use of biosecurity and therapeutic measures to help prevent SLD outbreaks.

## Introduction

Spotty Liver Disease (SLD) has been a persistent problem in the Australian and UK poultry industries for several decades and its presence in North America has recently been confirmed (1–3). In Australia, SLD was first noted in the 1980s (4, 5) and now the disease is regarded as one of the most important disease challenges for the Australian egg industry (6). Affected flocks can have an acute reduction in egg production of up to 25%, and an increase in mortality of up to 10% (1, 2). SLD outbreaks most commonly occur when the birds are reaching the peak of lay and the outbreaks can happen all year round (1, 4). The disease is particularly prevalent in free-ranging flocks but sporadically occurs in other housing systems such as conventional cages, controlled environmental cages, and barn systems (4).

Although SLD has been recognized for many years, perhaps as early as 1954 in the USA (7), the etiology of the disease was only determined recently, when a novel *Campylobacter* was isolated from infected birds in 2015 in England (8). Then, the same species was independently isolated in Australia from SLD affected birds and it was characterized and formally named as *Campylobacter hepaticus* in 2016 (9). SLD was induced by experimental exposure of egg-laying chickens to *C. hepaticus* and the bacteria was recovered from the birds; thus addressing Koch's postulates to formally demonstrate its role in the pathogenicity of SLD (10).

*Campylobacter hepaticus* is a Gram-negative, S-shaped bacterium, grows under microaerobic conditions at 37° and 42°C, and has single bipolar flagella. *Campylobacter hepaticus* ranges in size from 0.3 to 0.4 μm wide and 1.0–1.2 μm long (3, 8, 9). *Campylobacter hepaticus* has a reduced genome size (1.48–1.51 Mb) and a lower G+C content (27.9–28.5%) than most *Campylobacter* species (11, 12). *Campylobacter hepaticus* grows slowly *in vitro*; requiring at least 3 days for visible colonies to form on blood agar (3, 8). Isolation of *C. hepaticus* from primary sources is difficult because of the faster growth of other microorganisms and the absence of a fully selective media (3, 9). To date, all *C. hepaticus* isolates reported in the literature have been recovered from bile or liver samples, in which *C. hepaticus* is often present as a monoculture. Although *C. hepaticus* is present throughout the gastrointestinal tract of infected birds (13), no isolates have been recovered from such microbiological complex samples.

As *C. hepaticus* has only recently been identified, vaccines are yet to be developed to help to reduce the impact of SLD. Furthermore, laboratory studies attempting to control SLD using feed additives showed no reduction in disease. However, in field studies, both the incidence and the severity of SLD outbreaks can be reduced by the inclusion of some feed additives, particularly a combination of oregano and sanguinarine feed additives (14). There have been no published studies regarding the epidemiology of the bacterium and how it is transmitted between and within chicken flocks. Studies on closely related species commonly isolated from poultry, *Campylobacter jejuni* and *Campylobacter coli*, have shown the presence of these bacteria in multiple sources besides poultry (15–17), including, wild birds (18, 19), cattle (20), pigs (21), dogs (22), flies (23, 24), darkling beetles (25), water (26), and soils (27).

This study aimed to determine possible transmission routes of *C. hepaticus* in layer farms by investigating the presence of the bacterium in the birds and environmental samples, and by investigating the spread of infection within flocks. By defining potential sources of *C. hepaticus* and understanding the dynamics of spread to and within a flock, appropriate biosecurity measures can be designed to minimize transmission to and within flocks.

A large collection of fecal swabs, caecum, and bile samples were collected from pullets during rearing and from hens in production. A variety of environmental samples were also collected on farms, including soil, water, dust, wild bird feces, rats, mice, and insects. A selection of *C. hepaticus* isolates recovered from bile samples were subjected to whole genome sequencing to examine the conservation or divergence of *C. hepaticus* genomes in different outbreaks.

## Materials and Methods

### Study Design and Sample Collection

A total of 1,076 chicken and environmental samples were collected at 2–4 week intervals from three layer farms in Victoria and a further 764 chicken and environmental samples were collected from other chicken farms across Australia. For the three main study farms, the bird samples and environmental samples were collected when birds were transferred from rearing farm (16 weeks old) until peak laying period (26–30 weeks old). For samples from other farms across Australia, samples were collected from chickens across all ages, with or without clinical signs of SLD. All samples were transferred to the laboratory in insulated boxes with ice packs.

Samples were subjected to *C. hepaticus* specific PCR (SLD-PCR) and bacterial isolation to detect the presence of *C. hepaticus* in the samples. Genome sequencing and comparative genomic analysis were performed to examine the similarity of *C. hepaticus* isolates recovered from different farms.

### DNA Extraction

DNA from all samples was prepared using the DNeasy PowerSoil Kit (Qiagen) according to the manufacturer's instructions. DNA from fecal swabs and bile samples were prepared by either boiling of sample resuspended in water and direct use of the supernatant or DNeasy PowerSoil Kit as described previously (28). For each batch of DNA extractions cultured *C. hepaticus* cells were used as a positive control and water as a negative control.

### Polymerase Chain Reaction (PCR)

Isolated DNA was subjected to PCR amplification to detect the presence of *C. hepaticus* DNA. PCR primers specific to *C. hepaticus* were used as previously described by Van et al. (13). The PCR assay has been shown to be species-specific for *C. hepaticus*, with the limit of detection of the assay 10^0.9^ (7.9) CFU/reaction.

### Isolation of *C. hepaticus*

To isolate *C. hepaticus*, bile samples were directly streaked onto horse blood agar (HBA) plates [Brucella broth (BBL) supplemented with 1.5% agar (BBL) and 5% defibrinated horse blood (Equicell)], as described previously (9). A combination of filter membrane and *Campylobacter* selective media approaches (called the motile-filter method in this paper) were used for the isolation of *C. hepaticus* from fecal, caecal, and soil samples. Fifty milligrams of samples were resuspended in 200 μl sterile Milli-Q water and 50 μl of the mix was spotted onto 0.65 μm cellulose acetate filter membranes (Sartorius Stedim Biotech) and placed on the surface of HBA plates supplemented with *Campylobacter* selective supplement (Skirrow, Oxoid) (HBAS) and left for 30 min. The filter was then removed and the plate incubated. Motile organisms, including *C. hepaticus*, can move through the membrane whereas non-motile organisms are trapped on top and removed with the filter. Isolation of *C. hepaticus* from environmental samples was attempted by suspension of samples in Brucella broth (10 times dilution) and direct plating onto HBAS plates as well as using the motile-filter method. All plates were incubated at 37°C for 3–5 days under microaerobic conditions using CampyGen 3.5L (Oxoid, CN0035A) in an anaerobic jar.

### MALDI-TOF MS to Identify Bacterial Species

The identity of *C. hepaticus*–like colonies was confirmed by matrix assisted laser desorption/ionization time of flight mass spectrometry (MALDI-TOF MS) using a Microflex LT mass spectrometer (Bruker MALDI Biotyper System, Bruker Daltonics) according to the manufacturer's instructions.

### Whole-Genome Sequencing and Genomic Analysis

*Campylobacter hepaticus* isolates obtained from this study were subjected to whole-genome sequencing. DNA of *C. hepaticus* was extracted using an Isolate II Genomic DNA Kit (Bioline). The Nextera XT DNA Library Preparation Kit (Illumina) was used for genomic library construction and purified libraries were sequenced using Illumina MiSeq with 2 × 300 bp paired-end reads. Sequences were assembled using the A5 MiSeq pipeline (29) then annotated using RAST (http://rast.nmpdr.org/). Genome comparisons against the existing database of *C. hepaticus* sequences were performed using OrthoANI: https://www.ezbiocloud.net/tools/ani (ANI) and the Basic Local Alignment Search Tool (BLAST, https://blast.ncbi.nlm.nih.gov/Blast.cgi). Contigs with suspected plasmid elements and genes were Blasted against the NCBI database. Significant matches were identified with >98% coverage and identity to previously characterized plasmids from other *Campylobacter* species.

## Results

### Prevalence of *C. hepaticus* in Three Farms Monitored Over Time

In the three study farms from which samples were collected over time, a total of 1,076 chicken and environmental samples were analyzed (Table 1).

**Table 1:** The presence of *Campylobacter hepaticus* in three farms monitored over time.

**Sample type**	**No. samples**	***Campylobacter hepaticus*** **positive**							**SLD status**
		**PCR (positive/total)**						**Culture**	
**FARM 1**									
Age (weeks)		18	20	22	24	26	28		No *Campylobacter hepaticus* found during 10 weeks of study on Farm 1
Fecal swabs	348	0/95	0/47	0/57	0/50	0/50	0/49	Nd	
Bile	25		0/4	0/8	0/4	0/9		Nd	
Caecum	59	0/9	0/10	0/10	0/10	0/10	0/10	Nd	
Beetles	5	0/3	0/2					Nd	
Flies	4	0/1	0/3					Nd	
Rat feces	4	0/4						Nd	
Soil	34	1/1	0/18			0/15		Nd	
Mouse caecum	1	0/1						Nd	
**FARM 2**									
Age (weeks)		17	19	22	26	27	30		SLD outbreaks started to occur when chickens were 26 weeks old on Farm 2
Fecal swab	303	0/50	0/50	0/50	4/53	12/50	14/50	Nd	
Bile	19	0/5			2/3	4/10	1/1	3	
Caecum	13	0/5				8/8		Neg	
Litter	1		0/1					Neg	
Wild bird feces	1						1/1	Neg	
Rat feces	1						1/1	Neg	
Dust	3				2/2		1/1	Neg	
Water	1						1/1	Neg	
Flies	1			0/1				Neg	
Butterflies	1					0/1		Neg	
Earwig	1					0/1		Neg	
Spider	1					0/1		Neg	
**FARM 3**									
Age (weeks)		18	21	24	26	28			*C. hepaticus* was detected when chickens were at 24 weeks old, no SLD clinical signs. SLD outbreak occurred when chickens were 26 weeks old on Farm 3
Fecal swabs	235	0/45	0/50	3/50	9/40	23/50		Nd	
Bile	5			1/3		2/2		1	
Litter	1	0/1						Neg	
Rat feces	1					1/1		Neg	
Dust	3			1/2		1/1		Neg	
Beetles	4	0/1			0/2	0/1		Neg	
Flies	1					0/1		Neg	
Total	1,076								

*Nd, not done; Neg, negative; blank cell, samples not collected*.

On Farm 1, 432 chicken samples including fecal swabs, caecal content, bile, and 48 environmental samples were collected. No clinical signs of SLD and no *C. hepaticus* were detected during the 10-week period investigated in bird samples. However, one soil sample contained *C. hepaticus* DNA. Egg production and the mortality rate on this farm were normal. Egg production ranged between 92 and 94% from weeks 24 to 30.

On Farm 2, 335 chicken samples and 11 environmental samples were collected. No *C. hepaticus* was detected in samples taken at the time of transfer of the pullets from the rearing farm or in the first 4 weeks on the production farm (17, 19, and 22 weeks of age). However, at 26 weeks clinical signs of SLD were observed and confirmed on autopsy. Several samples from the layers began to register as *C. hepaticus* positive by PCR. At that time *C. hepaticus* was also detected by PCR in dust collected from the shed (2/2) and *C. hepaticus* was isolated from bile samples from birds with typical SLD lesions in their livers. However, the proportion of birds in which *C. hepaticus* could be detected in fecal swabs was low, with only 4 out of 53 samples *C. hepaticus*-PCR positive (Table 1). At the next collection times the proportion of PCR positive birds increased. At 27 weeks 12/50 and at 30 weeks 14/50 fecal swab samples were *C. hepaticus*-PCR positive. All environmental samples collected at 30 weeks on this farm, including wild bird feces, dust, rat feces and water were also PCR-positive for *C. hepaticus*. Egg production on this farm dropped from 93.4% at week 24 to 90.4% at week 26. After the low of egg production at week 26 it recovered to 93.7% by week 31. Mortalities peaked at week 26.

On farm 3, 240 chicken samples and 10 environmental samples were collected. All samples were PCR negative for *C. hepaticus* at 18 and 21 weeks old (the first two collection points). At the third collection point, when chickens were 24 weeks old, *C. hepaticus* was detected by PCR in 3/50 fecal swab samples, 1/3 bile samples and 1/1 dust samples, however, no clinical signs of SLD in chickens were observed. Two weeks later, when the birds were 26 weeks old, an SLD outbreak occurred on Farm 3 and *C. hepaticus* was detected by PCR in 23/50 cloacal swab samples, 2/2 bile samples, 1/1 rat fecal sample and 1/1 dust sample. Collected beetles and flies were negative for *C. hepaticus* in all three study farms (0/15). Egg production on this farm fell from 92.1% at week 24 to 87% at week 27 and unlike in Farm 2 egg production did not recover to 90% or above from week 30 onwards. Mortalities peaked at week 25.

*Campylobacter hepaticus* was isolated by culture from most of the bile samples from the birds with clear clinical signs of SLD. Failure to isolate was usually due to growth of other fast growing bacterial contaminants present in samples, which out-grew the slow-growing *C. hepaticus*. No *C. hepaticus* was cultured from any of the environmental samples.

### Prevalence of *C. hepaticus* in Various Farms Across Australia

In addition to the temporal study of *C. hepaticus* occurrence on three farms, samples were also taken on an *ad hoc* basis from other farms in Australia over a period of 1 year, collected by collaborating veterinarians during their routine farm visits. Samples were collected from birds in farms with and without SLD outbreaks.

A total of 710 chicken samples and 54 environmental samples were collected. *C. hepaticus* was detected in chicken samples and environmental samples (Table 2). No *C. hepaticus* was found in 1- or 2-week-old chickens but the bacterium was present in some pullets at 12, 17, and 18 weeks-old without any clinical signs of SLD. In one farm where *C. hepaticus* was detected in cloacal swabs by PCR at 18 weeks old, *C. hepaticus* was also found not only in cloacal swab samples but also in environmental samples including water, flies and rat feces. Eight weeks later, at 26 weeks old, an SLD outbreak occurred on this farm and *C. hepaticus* was again detected in environmental samples (mites and flies). *C. hepaticus* was found in some wild bird feces, rat feces and soil samples from these *ad hoc* samples. These types of environmental sources were also found to be positive for *C. hepaticus* in the main study farms in Victoria (Table 1). SLD outbreaks were mostly observed in laying hen when they were in peak production. However, one SLD outbreak was observed in chicken at 60–62 weeks-old, and *C. hepaticus* was isolated from these birds.

**Table 2:** The presence of *C. hepaticus* in *ad hoc* samples collected around Australia.

**Sample types/number of collections**	**Number of samples**	***C. hepaticus* PCR positive**	*C. hepaticus* culture positive^*^
**FECAL SWAB**			
<17 week/7	146	11	Nd
17–29 week/15	396	116	Nd
29–40 week/2	45	11	Nd
>40 week	0	0	0
**BILE**			
<17 week/1	4	0	0
17–29/27	100	40	14
29–40/3	3	2	2
>40/3	16	8	5
Rat feces/3	4	1	Neg
Wild Bird feces/2	5	1	Neg
Mites/3	3	2	Nd
Flies/4	6	3	Nd
Water /1	1	1	Neg
Soil/5	25	1	Neg
Beetles/1	2	0	Nd
Ants/2	4	Neg	Nd
Slatter bugs/1	1	Neg	Nd
Black flies/2	1	Neg	Nd
Beetles/1	1	Neg	Nd
Earwigs/1	1	Neg	Nd

* *Nd, not done; Neg, negative*.

Sixteen isolates from diverse locations (6 from Queensland, 6 from Victoria, 2 from Western Australia, 1 from South Australia and 5 from New South Wales) isolated during this study were subjected to whole genome sequencing.

### Isolation of *C. hepaticus* From Microbially Complex Samples

*Campylobacter hepaticus* has previously been isolated from bile or liver samples. Direct plating of such samples is usually successful because bile and liver samples from SLD affected birds often carry monocultures of *C. hepaticus* and are therefore not subjected to contaminant growth. However, environmental samples and samples from the gastrointestinal tract of chickens usually carry complex microbiotas, meaning the direct plating method or enrichment methods are ineffective. The motile-filter method was implemented and tested on fresh fecal samples from SLD affected birds, and *C. hepaticus* was recovered, thus demonstrating for the first time the successful isolation of *C. hepaticus* from microbiologically complex samples. The fecal samples used to demonstrate isolation from complex microbiota were from birds that were experimentally infected with *C. hepaticus*, as part of previously reported research (10). Once the motile-filter method was successfully tested it was applied to environmental samples, but no viable *C. hepaticus* could be recovered from any of the samples.

### Genome Sequencing of 16 *C. hepaticus* Isolates Showed That They Are Closely Related

Sixteen new isolates were sequenced and their genomes were compared to the reference genome sequence of *C. hepaticus* HV10 (GenBank: NZ_CP031611.1). As shown in Table 3, genome size ranged from 1,478,686 to 1,534,365 bp and the average G+C content ranged from 27.91 to 28.16%. The average nucleotide identity of the genome sequence of these isolates against *C. hepaticus* HV10 showed a high similarity of 99.95% amongst all isolates. The ANI values and MALDI TOF results identified the cultured isolates as *C. hepaticus*. Two isolates from Queensland (QLD) contain a plasmid with 99% identity to *Campylobacter jejuni* subsp. *jejuni* 81-176 plasmid, pTet, containing a tetracycline resistant gene, *tet(O)*. Phenotypic resistance to tetracycline was experimentally confirmed. The whole genome sequence data has been deposited in the NCBI database under BioProject PRJNA485661, the accession numbers of the individual genomes are detailed in Supplementary Information.

**Table 3:** The similarity of new *C. hepaticus* isolates compared to *C. hepaticus* type strain, HV10.

**Isolates/Location**	**Number of contigs**	**Genome size (bp)**	**% GC**	**OrthoANIu (%)**	**Plasmid**
*C. hepaticus* HV10/VIC	1	1,520,669	28.03	100	
VIC_1/VIC	43	1,481,639	27.91	100	
VIC_2/VIC	46	1,482,274	27.92	99.98	
VIC_3/VIC	44	1,484,585	27.91	99.98	
VIC_4/VIC	44	1,484,170	27.91	99.97	
VIC_5/VIC	42	1,482,790	27.91	99.99	
VIC_6/VIC	86	1,479,165	27.98	99.98	
QLD_1/QLD	46	1,517,443	28.00	99.96	Yes
QLD_2/QLD	45	1,521,414	28.00	99.95	Yes
QLD_3/QLD	47	1,522,162	27.97	99.96	
QLD-4/QLD	51	1,482,233	27.92	99.97	
QLD_5/QLD	49	1,478,686	27.93	99.96	
QLD_6/QLD	45	1,479,394	27.92	99.95	
WA_1/WA	49	1,534,365	27.94	99.97	
WA_2/WA	49	1,532,307	27.94	99.98	
SA_1/SA	32	1,524,262	27.97	99.98	
NSW_1/NSW	90	1,515,141	28.16	99.96	

## Discussion

Clinical SLD outbreaks were observed in two of the three farms that were monitored over 10 weeks. In both farms, SLD occurred during peak-laying age at 26 and 28 weeks of age; this timing agrees with previous reporting of the most common age at which disease is seen (4). Both outbreaks in the monitored farms occurred during winter. Outbreaks from other unmonitored farms occurred throughout the year. In previous decades the disease had also been referred to as “Summer Hepatitis” because of an apparent tendency to most commonly occur in summer. However, based on our findings from this epidemiological study and other experience over the last 5 years, it is clear that the disease can occur all year round. Although the first outbreaks of SLD in a flock generally occur as the birds enter peak lay, further outbreaks, within the same flock, can occur at later ages (4). In one of the *ad hoc* sampled flocks, an SLD outbreak occurred in birds of 60–62 weeks of age. *Campylobacter hepaticus* was successfully isolated from an SLD affected bird from this flock.

The identification of *C. hepaticus* as the etiological agent of SLD, and the development of sensitive PCR detection methods, has enabled us to move beyond the simple cataloging of clinical signs to now study the underlying dynamics of pathogen infection and spread. It is clear that birds can be infected with *C. hepaticus* many weeks before overt clinical disease becomes obvious. The *ad hoc* sampling showed birds as young as 12 weeks of age were infected, and this is of pivotal importance as it means rear pullets can be a source of contamination to a previously clean site. Furthermore, in the detailed temporal study, *C. hepaticus* was detected in Farm 3 two weeks before disease was seen. This has several implications. First, it indicates that infection with *C. hepaticus* may not be sufficient to induce disease; some other predisposing factors are also required. The hypothesized predisposing factor(s) may influence SLD outcomes by increasing the abundance of *C. hepaticus* or by altering the susceptibility of the host. Second, it shows that rapid molecular detection methods such as PCR can be used for early detection and identification of flocks at risk of an SLD outbreak. Forewarned of infection status, concerted efforts can be made to reduce the possibility of other predisposing events (e.g., changes in feed, problems with water supply) occurring, that could precipitate an SLD outbreak. Clinical observations have shown that birds are most likely to suffer the first occurrence of SLD when they enter peak lay (4). It has been hypothesized that the physiological changes that occur because of a rapid increase in egg production, such as negative nutrient balance, may affect liver metabolism and act as a predisposing factor for disease progression (6, 30). We hypothesis that factors affecting gastrointestinal tract microbiota during peak lay period, such as changes in feeding patterns, may play an important role in SLD development. These predisposing factors may act by increasing the population levels of the pathogen and/or by making the bird more susceptible to the as yet unknown virulence factors expressed by *C. hepaticus*.

In SLD affected farms, only 10–50% of birds had detectable levels of *C. hepaticus* in fecal swab samples. This demonstrated that some birds within a flock do not acquire the pathogen or are able to quickly overcome it during an SLD outbreak. It is not currently known if these birds have acquired immunity from previous exposure to *C. hepaticus* or whether they have just not been exposed to a sufficient infective dose. Based on this and other field observations and reliable induction by oral administration of large doses (10^8^ and more) of *C. hepaticus*, it has been surmised that infection is likely to spread within a flock via the fecal-oral route (6, 10). It has also been shown that birds manifesting clinical SLD have large numbers of *C. hepaticus* in their gastrointestinal tracts, (13), and in the current study it has been shown that viable bacteria can be cultured from the feces of infected birds. It, therefore, appears that all birds in a flock undergoing an SLD outbreak would be exposed to *C. hepaticus* via the fecal-oral route. However, the finding that not all birds in an infected flock have detectable levels of *C. hepaticus* may indicate that the infective dose of *C. hepaticus* is very high.

*Campylobacter hepaticus* DNA was detected in a variety of environmental samples including wild bird feces, flies, and rat feces from SLD-positive farms. These motile organisms might be vectors for *C. hepaticus* dissemination. A recent study that investigated biosecurity practices on Australian commercial layer farms showed that wild birds were commonly reported to be present in free-range farms (73%) and it was noted that they are potential sources of diseases that can be transmitted to laying hens (31). Flies have previously been implicated in the transmission of other *Campylobacter* species to chickens (32). Some water, soil, mite, and dust samples were also PCR-positive for *C. hepaticus* DNA. Water, soil, dust and mites could be intermediate sources for the transmission of *C. hepaticus* to and/or from the potential animal vectors and transfer to chickens and between chickens within a flock. Positive samples were mainly obtained from farms in which clinical SLD was occurring, but a few positive samples were from farms which had no apparent clinical SLD at the time of sample collection, indicating a potential route of transmission for *C. hepaticus* due to environmental factors described above. This may also explain the higher propensity of SLD in free-range layers, who have more frequent interactions with diverse environmental sources.

Although *C. hepaticus* DNA was detected in environmental samples by PCR (wild bird feces, rat feces, mouse caecum, soil, water, dust, mites, and flies), no viable *C. hepaticus* were recovered from these samples, even though the motile-filter method was used successfully for *C. hepaticus* isolation from chicken feces of experimentally infected birds. However, the fecal samples from the experimentally infected birds were qualitatively different to the environmental samples as they were collected immediately after birds defecated and were quickly (< 2 h) transferred to the laboratory, on ice, whereas the environmental samples were usually of unknown age and took considerable time (sometimes several days at ambient temperatures) to transport to the laboratory for analysis. *Campylobacter* spp. are usually considered to be sensitive to environmental exposure, so it is unsurprising that viable *C. hepaticus* could not be recovered. Given that PCR indicated many environmental samples were positive, these sources cannot be ruled out as initial transmission reservoirs. Another consideration is that various *Campylobacter* species are known to enter a viable but non-culturable (VBNC) state under stress, and therefore cannot be routinely recovered using conventional culture methods (33), which may also account for the discrepancies between the PCR positive and culture negative samples. It is possible that *C. hepaticus* VBNC cells could be present in environmental samples. VBNC cells can be resuscitated under specific conditions, such as ingestion (34), and so there may be bacteria present, that could cause infection in chickens, even though we haven't been able to detect them by conventional isolation methods. The available evidence indicates that *C. jejuni* VBNCs can be resuscitated by inoculation of chicken embryos but not by direct oral inoculation of chickens (35, 36), but *C. hepaticus* may be different. Further research is required to investigate this possibility and understand whether such organisms could play a role in disease transmission.

Genome sequencing and bioinformatics analysis of 16 isolates from different farms in VIC/QLD/WA/SA/NSW indicated that they are all highly similar to the *C. hepaticus* type strain, HV10. The genome sequences of all isolates were examined for plasmid content as plasmids may play an important role in dissemination of antibiotic resistance genes. Two isolates contained plasmids with very high sequence similarity to *C. jejuni* pTet-like plasmids. *C. hepaticus* isolates from Australia and UK have previously been reported to contain tetracycline-resistant plasmids, but they contained different *C. jejuni* and *C. coli* plasmids (11, 12). Currently, chlortetracycline is the main treatment option available for the control of SLD in Australia. With plasmid borne resistance arising in *C. hepaticus* the use of this treatment becomes ineffective in some flocks.

In conclusion, this study found that birds can be infected with *C. hepaticus* during rear and prior to the onset of lay, without any clinical SLD. SLD outbreaks occurred mainly at peak lay but could also occur earlier or later in the production cycle. Clean production sites may lose their negative *C. hepaticus* through the transfer of positive pullets. Therefore, it is of epidemiological significance to collect samples from birds from several weeks of age until the peak lay period to investigate when the birds become infected with *C. hepaticus*. Once birds are infected with *C. hepaticus*, measures, such as antibiotic treatment, have been used to recover from clinical SLD but the emergence of tetracycline resistance encoding plasmids foreshadows the need for alternative treatments and management practices. As environmental sources are a likely transmission source of *C. hepaticus*, biosecurity methods need to be strictly followed to prevent the spread of this bacteria, such as avoiding standing water on the range, as we found *C. hepaticus* can survive for several days in water (unpublished data). It appears that control of rodents and birds should also be emphasized as an important biosecurity measure to help reduce the probability of SLD outbreaks.

## Data Availability Statement

All datasets generated for this study are included in the article/Supplementary Material.

## Author Contributions

TV and RM conceived the study. CP, BV, and TV carried out the laboratory work. AA, TW, and PS collected samples and arranged for sample collection by other veterinarians. CP and TV drafted the manuscript and all authors edited the manuscript.

### Conflict of Interest

AA, TW, and PS are employed by the company Scolexia Pty Ltd. The remaining authors declare that the research was conducted in the absence of any commercial or financial relationships that could be construed as a potential conflict of interest.
