# Supplementary material for: Campylobacter hepaticus, the Cause of Spotty Liver Disease in Chickens: Transmission and Routes of Infection
Source: Front Vet Sci. 2020 Jan 15;6:505. doi: 10.3389/fvets.2019.00505 (PMC6974796; doi:10.3389/fvets.2019.00505)
Supplement: Supplementary file 1 [file Data_Sheet_1.DOCX]

| **SUBID** | **BioProject** | **BioSample** | **Accession** | **Organism** |
| --- | --- | --- | --- | --- |
| SUB6435512 | PRJNA485661 | SAMN13048811 | WHMC00000000 | *C. hepaticus* VIC_1/VIC |
| SUB6435512 | PRJNA485661 | SAMN13048812 | WHMD00000000 | *C. hepaticus* VIC_2/VIC |
| SUB6435512 | PRJNA485661 | SAMN13048813 | WHME00000000 | *C. hepaticus* VIC_3/VIC |
| SUB6435512 | PRJNA485661 | SAMN13048814 | WHMF00000000 | *C. hepaticus* VIC_4/VIC |
| SUB6435512 | PRJNA485661 | SAMN13048815 | WHMG00000000 | *C. hepaticus* VIC_5/VIC |
| SUB6435512 | PRJNA485661 | SAMN13048816 | WHMH00000000 | *C. hepaticus* VIC_6/VIC |
| SUB6435512 | PRJNA485661 | SAMN13048817 | WHMI00000000 | *C. hepaticus* QLD_1/QLD |
| SUB6435512 | PRJNA485661 | SAMN13048818 | WHMJ00000000 | *C. hepaticus* QLD_2/QLD |
| SUB6435512 | PRJNA485661 | SAMN13048819 | WHMK00000000 | *C. hepaticus* QLD_3/QLD |
| SUB6435512 | PRJNA485661 | SAMN13048820 | WHML00000000 | *C. hepaticus* QLD-4/QLD |
| SUB6435512 | PRJNA485661 | SAMN13048821 | WHMM00000000 | *C. hepaticus* QLD_5/QLD |
| SUB6435512 | PRJNA485661 | SAMN13048822 | WHMN00000000 | *C. hepaticus* QLD_6/QLD |
| SUB6435512 | PRJNA485661 | SAMN13048823 | WHMO00000000 | *C. hepaticus* WA_1/WA |
| SUB6435512 | PRJNA485661 | SAMN13048824 | WHMP00000000 | *C. hepaticus* WA_2/WA |
| SUB6435512 | PRJNA485661 | SAMN13048825 | WHMQ00000000 | *C. hepaticus* SA_1/SA |
| SUB6435512 | PRJNA485661 | SAMN13048826 | WHMR00000000 | *C. hepaticus* SW_1/NSW |

*Campylobacter hepaticus*, the cause of Spotty Liver Disease in chickens: Transmission and routes of infection.

Whole genome accession numbers.
